# Predictive value of phosphorylated neuro-filament h as a marker of brain injury after cardiac arrest

**DOI:** 10.1186/2197-425X-3-S1-A203

**Published:** 2015-10-01

**Authors:** S El Maraghi, H Saber, M El Saied, N Samir

**Affiliations:** Critical Care Department, Faculty of Medicine, Beni Suef University, Cairo, Egypt; Critical Care Department, Faculty of Medicine, Cairo University, Cairo, Egypt

## Intr

Anoxic brain insult is a sequalae of tissue hypoperfusion during cardiac arrest that may lead to varieties of neurological outcome in patients [1].One of the main drawbacks in the diagnosis of brain injury post-cardiac arrest is the absence of a widely available and rapid diagnostic test. pNFH might be an ideal biomarker for brain injury as it is axon specific [2],highly immunogenic [3] and an excellent target for antibody-based assays [4].

## Objective

The objective of our study was to assess whether pNF-H could provide useful diagnostic information about axonal injury in the early evaluation of such patients and whether levels of the pNF-H correlated with the outcome and prognosis of these patients.

## Methods

A total of 30 critically ill patients admitted to the critical care departments of Cairo and Beni Suef Universities and had cardiac arrest during their ICU stay were prospectively studied. Serum levels of pNF-H were assayed on day 1 and on day 3 post cardiac arrest. Neurofilament levels were correlated with Glasgow coma scale to assess conscious level on day 1 and on day 3 post arrest. Rankin score and cerebral performance categories (CPC) were used to determine patient outcome and the degree of disability.

## Results

pNFH levels showed a negative correlation with GCS on day 1 and day 3 in patients with brain injury post cardiac arrest (P= 0.001 & p < 0.001) respectively; hence higher pNF-H levels were associated with lower GCS on day 1 and day 3. There was a statistically significant positive correlation between pNFH and Rankin score on day 3 with r value = 0.814 & P value > 0.001. Patients with higher levels of pNFH on day 1 post arrest and on day 3 showed a greater CPC score hence liable for severe disability and poor outcome (CPC = 3,4,5), (r = 0.52, p = 0.003) and (r = 0.84, p < 0.001) respectively. The cut off level of pNF-H to detect severe disability and death was 0.201 ng/ml on day 1 with sensitivity 85% and specificity 70% with a p-value 0.022.

## Conclusions

pNFH is a promising diagnostic and prognostic marker in patients with brain injury post cardiac arrest. NFH levels can be used as a marker to detect the degree of disability and death in patients after cardiac arrest.
